# Music-Enhanced Emotion (Re)construction (MEER!): An Innovative Training Enriched with Participatory Live Music

**DOI:** 10.5334/pme.1692

**Published:** 2025-09-09

**Authors:** Johanna Schönrock-Adema

**Affiliations:** 1 University of Groningen and University Medical Center Groningen, Wenckebach Institute for Education and Training, Lifelong Learning, Education and Assessment Research Network (LEARN), Groningen, The Netherlands; 2 Hanze University of Applied Sciences, Lectorate Music in Context of the Research Centre Art & Society, Prins Claus Conservatoire, Groningen, The Netherlands

## Abstract

**Background and need for innovation:**

In healthcare, emotions are traditionally avoided as they might cloud clinical judgement. However, ignoring emotions may lead to emotional exhaustion and burnout, jeopardising quality of care. More attention to emotions is needed to support healthcare professionals’ wellbeing and vitality, especially given the high rates of emotional exhaustion and burnout among them and factors like workforce shortages, an ageing population and increasing workload demands.

**Goal of innovation:**

The training aims to support healthcare professionals’ wellbeing and vitality and help prevent emotional exhaustion and burnout.

**Steps taken for development and implementation of innovation:**

The training is grounded in the theory of constructed emotion, incorporates an evidence-informed pedagogical approach and uses participatory live music to teach healthy emotion construction. Based on the theory, we refer to this process as ‘emotion construction’ rather than ‘emotion regulation’. The training includes theoretical background, six exercises following a gradual build-up towards (re)constructing emotions, and homework assignments.

**Evaluation of innovation:**

Preliminary findings support music’s ability to evoke distinct memories, bodily sensations, feelings and emotions, corroborating its intended function. Eliciting personal memories with specific emotions through music supports the theory of constructed emotion and justifies its use in the training. First training evaluations included descriptions like enlightening, inspiring and empowering.

**Critical reflection:**

The training programme shows a clear build-up and alignment with the theory, while incorporating evidence-informed pedagogical steps seamlessly. Implementation challenges include obtaining funding and, due to time constraints of the target groups, implementing the full training, which we mitigated by developing variations.

## Background and Need for Innovation

It has been a longstanding belief in healthcare that emotions should be avoided or overcome as they might cloud clinical judgement. Consistent with this view, clinical practice and medical education primarily focus on medical knowledge, technique and science rather than emotional support, healthy engagement with emotions and humanism [1,2,3,4]. The prevailing medical culture values emotional detachment, affective distance and stress resilience [5,6,7,8]. However, ignoring emotions can negatively affect the work climate and jeopardise holistic, high-quality patient-centred care [1,9,10,11]. Modern insights suggest that neglecting emotions may harm healthcare professionals, leading to emotional exhaustion, empathy decline, compassion fatigue and burnout [5,7]. Strikingly, emotional exhaustion and burnout among healthcare professionals is a growing problem. With developments such as the growing workload demands due to the increasing elderly population, rising burden of chronic disease and multi-morbidity, increasing complexity of care and the shortage of healthcare personnel, this issue is expected to worsen.

The high rates of emotional exhaustion and burnout among healthcare professionals and the essential role of healthy engagement with emotions for (future) healthcare professionals’ wellbeing and functioning as well as for patient safety [1,5,12,13,14] signify the urgent need to prioritise a healthy emotional life. A pedagogical intervention that focuses on healthy engagement with emotions might benefit the self-care, vitality and wellbeing of healthcare professionals, which is crucial for high-quality patient care. With ‘healthy engagement with emotions’ I mean reducing unnecessary or unjustified negative emotions and emotional suffering that hinder well-being and functioning and, instead, constructing more appropriate and healthier emotions that promote the overall emotional well-being, resilience, mental health and effective functioning of healthcare professionals. According to Barrett’s theory of constructed emotion [15], emotions are constructed by the brain through predictions where bodily sensations are linked to the context and past experiences. As emotions can also be influenced by, for instance, bodily sensations that are not directly related to the current situation, creating understanding and awareness of such instances may help in constructing healthier emotions.

## Goal of Innovation

This project aimed to develop an innovative training to teach (future) healthcare professionals how to engage healthily with their emotions and cultivate healthy emotion (re)construction. Ultimately, this training seeks to foster self-care, wellbeing, meaningful social connections and interactions, and a safe, positive learning and working environment, and to enhance patient safety.

## Steps Taken for Development and Implementation of Innovation

The innovative aspects of the training include (1) a relatively new theory as a training foundation; (2) a recently published pedagogical approach to guide the training process; and (3) participatory live music to elicit memories, feelings and emotions, providing a rich method for participants to tune in to, engage with and (re)construct their emotional experiences.

### Theoretical framework

Barrett’s theory of constructed emotion forms the theoretical foundation of the training [15]. This theory posits that emotions are not innate, fixed responses but are constructed by our brain. Barrett argues that emotion and cognition cannot be seen as separate entities because they interact in the process of constructing emotional experiences. She proposes the term ‘emotion construction’ to describe how emotions are actively created based on various factors, rather than ‘emotion regulation’ which focuses on managing existing emotions. Accordingly, this article uses the term ‘construction’ instead of ‘regulation’. According to Barrett, our brain’s most important task is keeping our body alive, so we survive and thrive [15].^1^ To do so, our brain constantly makes predictions based on past experiences, context and physiological states. The emotions we experience or perceive in others are also predicted or constructed by our brain. This construction of emotions involves predictive coding, where the brain generates multiple competing simulations based on past experiences, context and physiological states to determine what new sensory input is most like. These simulations act as prediction models that continuously anticipate and interpret sensory input and develop our emotional responses accordingly. Thus, our brain constructs emotions by linking our bodily sensations to the context using prior experience.

Following Barrett’s theory, we can influence our emotions by adjusting our experiences and predictions. By changing today’s experience, we can change tomorrow’s predictions and transform our emotional lives. Changing these predictions can be done by altering the ingredients that make up for the predictions, such as changing our bodily sensations or reinterpreting our bodily sensations by linking them to different memories or based on an analysis of the situation that led to identifying factors influencing these sensations or the simple feelings connected to them. Constructing healthy emotions is positively related to having a more granular or detailed emotion vocabulary [16]. In other words, if we can specify our emotions in more detail, we are better able to effectively construct healthy emotions.

Learning to construct healthy emotions requires (1) becoming aware of bodily sensations, feelings and emotions, (2) understanding their interrelationship and (3) their relation to prior experiences and context, (4) expanding or refining the existing emotion vocabulary to become better able to specify emotions, and (5) experimenting with changing the experience and (re)constructing healthy emotions rather than suppressing unhealthy ones [15,17]. Thus, healthy emotion (re)construction may foster healthcare professionals’ self-care, affective functioning, overall wellbeing, resilience and social interactions, [1,5,7,9,10,11,12,13,14,16]. By improving healthcare professionals’ emotional health, they are better equipped to engage empathetically and effectively with patients, thereby promoting patient-centred care [1,5,7,12,13,14] and strengthening the healthcare system as a whole.

### Pedagogical approach

The evidence-informed pedagogical approach of Fleer et al. was applied in this training [18]. Originally developed for professional identity development in undergraduate education, it was explicitly portrayed as a generic approach easily transferable to other educational settings. The approach incorporates fundamental pedagogical principles translated into a six-step reflective practice structure which is very suitable for the MEER! training. Translated to this context, the steps are: (1) have an emotion-related experience; (2) observe emotional responses to the experience; (3) externalise reflections on these responses (e.g., through drawing or writing); (4) share reflections with peers; (5) broaden trainees’ perspectives on emotion construction; (6) explore options to construct healthy emotions through experimentation. According to Fleer et al. [18], consistently and repeatedly using these steps in reflective practices helps trainees gradually internalise them. Such internalisation may foster a reflective attitude that enables healthy engagement with future emotions. Importantly, the sequence of these steps is flexible [18] and not every exercise or training component needs to include all six steps, as long as all steps are integrated throughout the entire programme (personal communication with Fleer 2024).

### Participatory live music approach

The MEER! training uses a person-centred participatory live music approach to support the learning and development of healthy emotion (re)construction. This approach tailors the music and live improvisations to the specific context, experiences, needs and feelings of individual trainees. ‘Participatory’ refers to the active involvement of participants in selecting or conceiving the performed music, ensuring that the musical experiences are relevant and impactful. Inspired by the positive outcomes of live music in patient care [19,20], this method involves experienced musicians who are familiar with this type of music making. In the training, participatory live music is used to elicit memories, feelings and emotions, providing a rich method for trainees to engage with and (re)construct emotions. The goal is to emotionally engage trainees and help them experience and reflect on authentic emotions so they can work with them and learn to construct healthy emotions.

### Resulting programme

The programme resulting from these ‘building blocks’ includes, besides theoretical background, six exercises gradually building up towards (re)constructing emotions, and homework assignments linked to exercises 2–6.^2^

*Exercise 1: Musical resonance*. This exercise aims to acclimatize participants to the setting, build safety^3^, mutual understanding and appreciation within the group, and explore physical reactions, feelings, emotions, images, memories and interpretations induced by music, and emotional resonance with music. Musicians improvise three pieces to evoke different emotions (pedagogical step 1). Students observe (pedagogical step 2) and externalise (pedagogical step 3) their physical, mental and affective reactions to the musical experience. Then they form groups based on the piece resonating most with them, share their experiences (pedagogical step 4) and compare their feelings, thoughts and the memories evoked by the music. Subsequently, they share and discuss their main observations, experiences, reflections and insights in the whole group (which may imply pedagogical step 5). These pedagogical steps are similarly applied in the following exercises.

*Exercise 2: Musical elements and physical sensations*. This exercise aims to help participants recognise how different musical elements evoke physical sensations, reflect on and specify bodily signals and become aware of the unique nature of each individual’s physical experiences and interpretations. Musicians improvise, varying musical elements to evoke different physical sensations. Students observe and externalise their physical sensations, share and discuss their experiences in groups and, subsequently, share the main outcomes of their groups’ discussions in the whole group where their perspectives may be broadened by referring to general insights and literature (pedagogical step 5).

*Exercise 3: Dissecting feelings*. This exercise aims to map feelings based on pleasantness (valence) and intensity (arousal) and to become aware of the unique nature of each individual’s physical and affective experiences and interpretations. Musicians ask trainees about emotionally significant past experiences without specifically asking about valence, arousal or emotions. They then improvise at different valence and arousal levels. Trainees observe what they themselves experience and note their feelings on a valence-arousal model before sharing in groups. This exercise helps participants understand the degree of pleasantness (valence) and intensity (arousal) of their feelings and recognise individual differences in these experiences.

*Exercise 4: Feelings and emotions*. This exercise focuses on experiencing and becoming aware of emotions, understanding the link between emotions and physical sensations and developing a more granular emotion vocabulary. Therefore, input regarding emotionally significant past experiences is gathered from the trainees after recalling such experiences in small groups. Musicians ask trainees about the emotionally significant past experiences retrieved and improvise based on these moments or situations. Trainees observe their emotions, externalise the initial emotion they experience, share their experiences in groups and explore, using emotion tools, any emotions that might also apply to their experience to identify the emotion word that most correctly reflects what they experienced. This exercise helps participants broaden and refine their emotional vocabulary.

*Exercise 5: Experimenting with healthy emotion (re)construction*. This exercise focuses on experimenting with and transforming emotions by linking physical sensations to different affective feelings and emotions through contextual analysis and/or visualisation. Trainees choose a recent memorable moment where they struggled with emotions, e.g., experiencing inappropriate emotions or emotions that disrupted their balance. These situations are discussed in small groups and then in the whole group with the trainers to clarify the context, physical and affective feelings, thoughts and desired emotions. Musicians improvise music that fits these experiences, allowing trainees to experiment with linking healthier emotions to the same physical sensations based on the preceding discussion (pedagogical step 6). This experimentation can be based on visualising similar situations which went along with other emotions or on identifying and peeling-off contextual factors that unjustly caused unhealthy emotions, such as lack of sleep, insufficient rest, hunger or cold. Such circumstances may have negatively affected bodily sensations and the accompanying simple feelings and, consequently, the construction of emotions. Peeling-off such aspects allows trainees to remove noise and better specify appropriate emotions for the actual situation, thereby gradually replacing distorted, unnecessarily unhealthy emotions with healthier, more specific and adequate ones. Subsequently, trainees reflect on their experience regarding reinterpreting physical sensations through contextual analysis, re-labelling and visualisation.

*Exercise 6: The power of positive emotions*. This exercise is comparable to exercise 4 but solely focused on positive emotions. It aims to make participants aware of how positive feelings and emotions contribute to positive outcomes and to understand the link between these emotions and physical sensations. Trainees choose a recent memorable moment where they experienced positive emotions, leading to success or deep satisfaction. The situations are, preferably, recognisable to all trainees. Musicians improvise music based on these experiences. Trainees observe and externalise their emotions, share these in groups and explore – again using an emotion tool – any emotions that might apply to their experience, trying to identify the emotion word that most correctly reflects what they experienced. This helps them understand the relationship between physical sensations and emotions and to develop a more granular emotion vocabulary.

The training programme was facilitated by two trainers experienced in guiding students and delivering educational content, including the developer of the training, and three professional musicians who played live music. The participants were asked to prepare for the workshop by watching a TED Talk by Barrett (see footnote 1). When the participants entered the room, the musicians played a couple of music pieces to create a warm and welcoming atmosphere. After welcoming the participants, the trainers conducted a short introduction activity where they presented some questions to get to know the participants, including questions about emotions and music. Then the participants were introduced to the theory and to the programme of the training, to clarify the structure and rationale behind it, before proceeding with the exercises.

## Evaluation of Innovation

We performed a pilot study in which we conducted a workshop to test and evaluate training elements. All 14 participants, including teachers, healthcare professionals and education researchers, completed a feedback form, and 13 participants voluntarily returned their completed hand-outs, providing material that allowed us to check whether the exercises achieved their intended outcomes. Additionally, some participants approached us after the training to compliment us or share additional thoughts. Furthermore, we used our own experiences and observations to improve the training. Our evaluations and observations yielded the following preliminary findings.

In exercise 1, three pieces of music – differing in style, emotional tone and atmosphere – were played: Elvis Presley’s *Can’t help falling in love*, an Irish Jig and an abstract improvisation. The Elvis piece predominantly brought about feelings of calmness, relaxation and reflection. Participants listening to the Elvis Presley piece reported bodily sensations, feelings and emotions like relaxed breathing, a sense of peace, comfort, carefreeness, relaxation, a sense of belonging, confidence, tenderness and inner peace, but also deeper emotional resonance linked to personal memories, with bittersweet feelings associated with a loved one’s funeral, like nostalgia or melancholia. In contrast, the Irish Jig, characterised by its lively and energetic nature, made participants feel a strong desire to move and dance, with physical sensations of increased energy and engagement. This piece, consisting of two parts, started sparking curiosity and anticipation, even some disturbance, quickly transforming into joy, happiness, optimism, energy and a celebratory mood, as well as a sense of community and togetherness. The third piece, an improvisation, stood out for its dynamic and unpredictable qualities, eliciting mixed physical sensations of tension and relaxation, reflecting the evolving and unpredictable nature of the music. It evoked a wide range of emotions from confusion and suspense to connection, but also frustration, disturbance, disengagement, ominousness and even worry and anxiety. In sum, the three pieces evoked clearly distinct memories, bodily sensations, feelings and emotions, thus providing support for the intended effects of music and underscoring the rationale for using music in the training. Additionally, the song evoking memories and emotions of a loved one’s funeral supports Barrett’s theory that emotions are constructed by linking bodily sensations to context and past experiences.

In exercise 4, two pieces of music were improvised based on the input of participants. As this was a mixed group of participants not all working in patient care, the input was rather general so that all participants could identify with it: one inspired by the excitement on the day before ‘Sinterklaas’, a traditional Dutch celebration with presents that traditionally brings a lot of excitement in children in the preceding weeks, and one inspired by the turmoil in society due to the threat of cuts in the education and education research budgets. Four emotion tools, each displaying a range of emotions to help participants identify and specify their emotions, were available and used during the workshop. The evaluation showed a tendency towards favouring the tools with the most emotion alternatives. After the session, one of the participants explicitly mentioned being more able to put emotions into words due to the exercise, which supports the aim of the exercise, namely extending or refining the existing emotion vocabulary. As such, we obtained preliminary support for the effectiveness of exercise 4.

At the end of the workshop, participants summarised their experience in one word, with the responses including “interesting”, “exciting”, “enlightening”, “inspiring”, “useful”, “empowering”, “inventive”, “fun” and “leaves you wanting more”. Additionally, one participant found it difficult to express their experience, which may have been an indication that it was hard to put the impact of the training into words. One participant also complimented that the training made the theory come to life.

## Critical Reflection on Process

Reflecting on the development process, several successes and pitfalls were noted. A main success was the alignment of the training programme with Barrett’s theory of constructed emotion [15] and the pedagogical steps outlined by Fleer et al. [18]. To achieve this alignment, the programme deliberately follows a build-up from ‘getting out of your head’ to becoming aware of bodily sensations and focusing on how these bodily sensations translate into simple feelings. It then builds towards experiencing emotions and increasing the granularity of trainees’ emotion vocabulary. Thus, the programme aims to help participants understand the relationship between bodily sensations, simple feelings and emotions, the link of emotions with context and past experiences, to create insight that similar bodily sensations can be linked to different emotions, and to broaden and refine their emotion vocabulary as the (re)construction of healthy emotions is more effective with a more granular emotion vocabulary. Subsequently, the programme moves to analysing aspects related to emotional situations, including bodily sensations, context and past experience, to culminate in experimenting with changing the current experience and constructing healthier emotions based on the outcomes of the analysis and on visualisation.

Throughout the training, Fleer et al.’s [18] pedagogical steps are integrated seamlessly. Participants undergo emotion-related experiences, observe their responses, externalise and share their reflections, broaden perspectives and explore freedom of choice through experimentation.

The theoretical foundation and evidence-informed pedagogical approach ensure participants can engage with and construct their emotions effectively, in order to attain improved emotional well-being and resilience.

During the development process, various workshops were held to translate the existing participatory live music approach to the emotion (re)construction training setting. This approach was effective for clearly articulating and refining the rationale for developing the training, its aims and foundations, and for experimenting with the music practice. Additionally, the workshops provided a breeding ground for developing the first exercise. The workshops also led to many conversations with participants involved in developing professional development courses, which further helped to articulate and structure thoughts and brainstorm about the training. Piloting the workshop proved very effective for developing the supervisor’s guide.

However, there are some implementation challenges. The costs of hiring professional musicians can be a challenge. Fortunately, funding is currently covered by an innovation prize and, in our context, junior doctors in postgraduate training have a budget for training. Another challenge is implementing the training as intended, given the limited time available for the target groups and the emphasis in clinical practice and (continuing) medical education on medical knowledge, technique and science. To address this challenge, several variations of the ideal workshop format – five weekly 2-hour sessions – were developed. These variations include single sessions of 1.5, 2 and 3 hours, as well as two 4-hour sessions, to accommodate different time constraints. While these adjustments mean the full training cannot be given, preventing trainees from reaching the core practice of changing their experience, they do allow participants to engage with the training according to their schedules. This flexibility enables us to reach and serve (future) healthcare professionals who otherwise might not have participated. Applying these formats offers us the opportunity to gain experience with training elements and improve our understanding of them. This will help us enhance instructions and exercises, determine when and how to broaden trainees’ perspectives, tailor the training to different target groups and enhance the training overall. Additionally, applying different formats enables us to investigate first impressions and effects of the training, which may help us build support and recognition for its importance and value. Gaining recognition is crucial for balancing the focus on medical knowledge, technique and science with attention to a healthy emotional life. Advocating for this shift is vital for fully integrating the training into curricula, continuing medical education or human resource training plans. Fortunately, there is a growing call for more attention to emotions in medical education. This increasing awareness of the importance of emotional support, healthy engagement with emotions and humanism in healthcare is promising for transforming healthcare education and practice and generating more emphasis on healthcare professionals’ self-care, wellbeing and vitality. The conscientious development of this innovative training and promising initial experiences support its potential to impact the field significantly and support healthcare professionals’ affective functioning, overall wellbeing and resilience, and hence their vitality and sustainable employability, as well as high-quality patient care.

## Footnotes

Also see https://ideas.ted.com/try-these-two-smart-techniques-to-help-you-master-your-emotions (accessed 10 January 2025).

Those interested in more detailed information about the exercises and their implementation are welcome to contact the author.

To further ensure a safe and supportive environment, we took several measures, including asking participants to keep all experiences shared during the sessions confidential, making explicit that sharing is voluntary, forming small groups of participants to create a safe space and facilitate participation, and advising participants to avoid sharing experiences that might be too heavy or emotionally intense.

## References

[1] Satterfield JM, Hughes E. Emotion skills training for medical students: a systematic review. Med Educ. 2007;41(10):935–41. DOI: 10.1111/j.1365-2923.2007.02835.x 17822414

[2] Brodahl KØ, Storøy HLE, Finset A, Pedersen R. Medical students’ experiences when empathizing with patients’ emotional issues during a medical interview – a qualitative study. BMC Med Educ. 2022;22(1):145. DOI: 10.1186/s12909-022-03199-9 35246126 PMC8895666

[3] Coulehan J, Williams PC. Vanquishing virtue: the impact of medical education. Acad Med. 2001;76(6):598–605. DOI: 10.1097/00001888-200106000-00008 11401802

[4] Rieffestahl AM, Morcke AM, Mogensen HO, Risør T. When the patient is absent in patient-centered communication training: a discursive analysis of how medical students learn to interact with patients. Teach Learn Med. 2024:36(3);269–79. DOI: 10.1080/10401334.2023.2217169 37266998

[5] Shapiro J. Perspective: does medical education promote professional alexithymia? A call for attending to the emotions of patients and self in medical training. Acad Med. 2011;86(3):326–32. DOI: 10.1097/ACM.0b013e3182088833 21248595

[6] Blasco PG, Moreto G, De Benedetto MAC. Educating Physicians for the Aging World: A Humanistic Approach in Doctoring. In: Demurtas J, Veronese N, editors. The Role of Family Physicians in Older People Care. Practical Issues in Geriatrics. Switzerland: Springer Nature Switzerland; 2022. pp. 421–49. DOI: 10.1007/978-3-030-78923-7_26

[7] Karnieli-Millera O, Palombo M, Laor N. The hidden curriculum of breaking bad news: Identification of three dimensions and four communication patterns. Patient Educ Couns. 2023;114:107807. DOI: 10.1016/j.pec.2023.107807 37236123

[8] Ajjawi R, Olson RE, McNaughton N. Emotion as reflexive practice: A new discourse for feedback practice and research. Med Educ. 2022;56(5):480–8. DOI: 10.1111/medu.14700 34806217 PMC9299671

[9] Correa-Rivero H. What did 3 years of medical humanism leave in me? Feelings, judgments and experiences narrated by medical students. Medicon Med Sci. 2022;2(4):3–10. DOI: 10.55162/MCMS.02.025

[10] Eichbaum QG. Thinking about thinking and emotion: the metacognitive approach to the medical humanities that integrates the humanities with the basic and clinical sciences. Perm J. 2014;18(4):64–75. DOI: 10.7812/TPP/14-027 25662528 PMC4206174

[11] Moyer CA, Arnold L, Quaintance J, Braddock C, Spickard A III., Wilson D, Rominski S, Stern DT. What factors create a humanistic doctor? A nationwide survey of fourth-year medical students. Acad Med. 2010;85(11):1800–7. DOI: 10.1097/ACM.0b013e3181f526af 20881828

[12] Kearney MK, Weininger RB, Vachon ML, Harrison RL, Mount BM. Self-care of physicians caring for patients at the end of life: “Being connected … a key to my survival.” JAMA. 2009;301(11):1155–64. DOI: 10.1001/jama.2009.352 19293416

[13] Tanriover O, Peker S, Hidiroglu S, Kitapcioglu D, Inanici SY, Karamustafalioglu N, Gulpinar MA. The emotions experienced by family medicine residents and interns during their clinical trainings: a qualitative study. Prim Health Care Res Dev. 2023;24:e25. DOI: 10.1017/S1463423623000051 37016917 PMC10130843

[14] Ardenghi S, Russo S, Bani M, Rampoldi G, Strepparava MG. The role of difficulties in emotion regulation in predicting empathy and patient-centeredness in pre-clinical medical students: a cross-sectional study. Psychol Health Med. 2023;28(5):1215–29. DOI: 10.1080/13548506.2021.2001549 34753373

[15] Barrett LF. The theory of constructed emotion: an active inference account of interoception and categorization. Soc Cogn Affect Neurosci. 2017;12(1):1–23. DOI: 10.1093/scan/nsw154 27798257 PMC5390700

[16] Hoemann K, Barrett LF, Quigley KS. Emotional granularity increases with intensive ambulatory assessment: methodological and individual factors influence how much. Front Psychol. 2021;12:704125. DOI: 10.3389/fpsyg.2021.704125 34393942 PMC8355493

[17] Shapiro S. Mindfulness practices for challenging times: emotion regulation, shifting perspective, compassion for empathy distress. Altern Complement Ther. 2020;26(3):109–11. DOI: 10.1089/act.2020.29277.ssh

[18] Fleer J, Smit MJ, Boer HJ, Knevel M, Velthuis F, Trippenzee M, De Carvalho Filho MA, Scholtens S. An evidence-informed pedagogical approach to support professional identity formation in medical students: AMEE Guide No. 171. Med Teach. 2025;47(4):580–8. DOI: 10.1080/0142159X.2024.2387809 39110856

[19] Van der Wal-Huisman H, Groen H, Heineman E, Van Leeuwen BL. The Effect of Live Bedside Music on Pain in Elderly Surgical Patients. A Unique Collaboration. OBM Geriat. 2020;4(3):125. DOI: 10.21926/obm.geriatr.2003125

[20] Van der Wal-Huisman H, Van den Berg NM, Paans W, Bezold L, Stegemann T, De Graeff P, Van Leeuwen BL. Live bedside music for hospitalized older adults: A qualitative descriptive interview study. Int J Older People Nurs. 2023;18:e12574. DOI: 10.1111/opn.12574 37731184

